# Late diagnosis of human immunodeficiency virus infection is linked to higher rates of epilepsy in children in the Eastern Cape of South Africa

**DOI:** 10.4102/sajhivmed.v21i1.1047

**Published:** 2020-06-30

**Authors:** Isabel A. Michaelis, Maryke Nielsen, Craig Carty, Markus Wolff, Caroline A. Sabin, John S. Lambert

**Affiliations:** 1 Department of Health, Faculty of Paediatrics, Walter Sisulu University, Mthatha, South Africa; 2 Department of Paediatrics and Child Health, Faculty of Infectious Disease, Malawi-Liverpool-Wellcome Clinical Research Facility, Blantyre, Malawi; 3 Institute of Infection and Global Health, Faculty of Clinical Infection, Immunology and Microbiology, University of Liverpool, Liverpool, United Kingdom; 4 Department of Paediatrics, Queen Elizabeth Central Hospital, Blantyre, Malawi; 5 Department of Evidence-Based Social Intervention, Faculty of Sociology, University of Oxford, Oxford, United Kingdom; 6 Department of Neuropaediatrics and Social Paediatrics, Faculty of Paediatrics, Vivantes Klinikum Neukolln, Berlin, Germany; 7 Department of Medical Statistics and Epidemiology, Faculty of Population Health Sciences, University College London, London, United Kingdom; 8 Department of Infectious Diseases, Faculty of Infectious Diseases and Genitourinary Medicine, UCD School of Medicine, Dublin, Ireland

**Keywords:** epilepsy, HIV-infection, children, WHO staging, paediatric ART data management tool

## Abstract

**Background:**

Human immunodeficiency virus (HIV)-positive children may present with a wide range of neurological disorders. Among these, epilepsy is of key concern because of its lifelong impact and potential for damage to the central nervous system (CNS). Few studies in developing regions have investigated the prevalence and aetiology of epilepsy in HIV-infected children as a key population.

**Objectives:**

We describe the prevalence of epilepsy, associated neurological disabilities, immunological status, clinical stage and history of CNS infection at epilepsy diagnosis in a cohort of HIV-infected children receiving antiretroviral therapy (ART) in the Eastern Cape of South Africa.

**Methods:**

We conducted a retrospective study (2004–2014) at two major referral sites for HIV-infected children diagnosed with epilepsy aged 0–16 years. Eligible subjects were extracted from the electronic medicine bridging access to care in excellence (EMBRACE) Paediatric Cohort using the Paediatric ART Data Management Tool (PADMT). Fixed data fields were interrogated for exposures to antiepileptic drugs. Unstructured ‘comments’ fields were searched for the terms: epilepsy, seizures, fits and szs, as well as abbreviated versions of common antiepileptic drug names. Eligible subject folders were then retrieved to validate the digital data.

**Results:**

From 2139 children enrolled in the two sites, 53 children were diagnosed with epilepsy (2.48%). In these, the median CD4 count was 591 cells/mm^3^, and the mean viral load was 4.9 log copies/mL, with undetectable viral loads in only seven children (14.0%). World Health Organization (WHO) clinical HIV stage was available for 46 patients of the sample, with 3, 6, 26 and 11 children graded at stages 1, 2, 3 and 4, respectively. Forty percent children had a history of CNS infection prior to the epilepsy diagnosis, and 55% children were reported to have school problems.

**Conclusions:**

In this descriptive study, the prevalence of epilepsy among children with HIV was 2.48%, mostly diagnosed in advanced HIV-disease stages. Our findings support the usefulness of early detection and initiation of ART in HIV-infected children in order to reduce the risk of epilepsy. In addition, our study demonstrates that novel techniques are effective in accessing cohort-level data that allow interrogation of both structured and unstructured clinical data.

## Introduction

The latest World Health Organization (WHO) report on human immunodeficiency virus (HIV) and acquired immunodeficiency syndrome (AIDS) estimates that 3.2 million children are infected with HIV worldwide, of whom 91% are living in sub-Saharan Africa.^1^ South Africa is regarded as having the largest HIV and AIDS burden in the world, with an estimated 17.6% of the population living with the disease; between 370 000 and 450 000 children younger than 14 years in South Africa are thought to be infected with the virus.^1^

Human immunodeficiency virus-related neurological manifestations are common in both adults and children.^2,3,4,5,6^ They include opportunistic central nervous system (CNS) infections, behavioural problems and psychiatric disorders, as well as neurodevelopmental delay and epilepsy.^4^ Epilepsyis a potentially disabling disease whose treatment is challenging in sub-Saharan Africa,^7,8,9,10,11,12,13^ particularly among children with HIV infection, who are often orphaned and have to face poverty.^14^

The prevalence of seizures in children with confirmed HIV infection has been reported to range from 2% to 14%,^9,15,16,17,18^ and the prevalence of epilepsy is estimated between 0.29% and 11.3%.^19,20,21^ This wide variation is believed to be a result of differences of risk factors in different regions.^22^

Epilepsy in HIV-infected persons may occur as a result of several different processes: direct viral damage to the brain by uncontrolled viral replication; following a CNS infection by opportunistic pathogens; as a result of malignancies; following the use of some medications; as a result of metabolic and electrolyte derangements; or as a secondary acquired pathology because of HIV encephalopathy.^4^

Of interest, Bearden et al.^19^ argued that earlier initiation of antiretroviral therapy (ART) might prevent the manifestation of epilepsy in children with HIV infection. This study was conducted in Botswana, and there is no other study on record so far to confirm or challenge these results in a different population.

We describe the prevalence of epilepsy, including associated neurological disabilities, immunological and clinical status and history of CNS infection in children with HIV infection on ART in a mixed rural/urban area in the Eastern Cape. The aim is to show that late diagnosis of HIV infection and delayed initiation of ART in children are linked to higher rates of epilepsy.

## Material and methods

### Setting

We conducted a retrospective study (2004–2014) at two major referral sites for HIV-infected children aged 0–16 years in the Eastern Cape, South Africa. One centre is a paediatric and adolescent HIV referral clinic in a tertiary hospital in East London, and the other is the HIV clinic for children and adolescents in the township of Mdantsane, which serves as a major referral centre as well as the local health clinic.

The Eastern Cape belongs to one of the most underprivileged parts of South Africa, and its health service is among the most under-resourced. Together, the two hospitals serve around 3.5 m people, including more than 890 000 children younger than 15 years (Census 2011 Municipal report).

In South Africa, ART became available in state health facilities from 2004. At this time, the criteria for ART required that children had advanced HIV disease, assessed by WHO staging.^23,24^ The eligibility criteria for initiation of ART and the type of first-line regimen for infants and children with HIV infection following HIV guidelines changed in 2004, 2010, 2013 and 2015.^25^ The clinicians in our setting used the appropriate guidelines for the specific year the children were firstly seen in the clinics. Since 2014, every child younger than 5 years with confirmed HIV infection was eligible for treatment, regardless of his/her clinical (WHO staging) and laboratory status (CD4 counts)^26^; since 2017, all people in South Africa with confirmed HIV infection have immediate access to ART.

In our setting, the routine approach to children presenting with seizures consists of a detailed clinical history and physical examination, including blood pressure, as well as assessment of blood glucose and, if indicated, antiepileptic drug plasma levels. Emergency computed tomography (CT) is performed if the child has new-onset focal seizures, encephalopathy or prolonged neurological fallout. In children with suspected meningitis, encephalitis or other inflammations, appropriate serum markers are investigated, and a lumbar puncture is performed unless contraindicated. Human immunodeficiency virus testing is offered to all children. In children known to be HIV infected, CD4 count and HIV viral loads (VLs) are assessed.

Cases were identified by screening all available subfiles for ‘epilepsy’ and by interrogating the fixed data fields of an electronic database, the Paediatric ART Data Management Tool (PADMT), for exposure to antiepileptic drugs. Unstructured ‘comments’ fields were hand-searched for relevant terms like e*pilepsy, seizures, fits* and szs, *paroxysmal events, faints* or *syncope* or electroencephalogram (EEG*)*. Eligible subject folders were then retrieved to validate the digital data.

Children with a single seizure or single seizure episode during acute illness because of metabolic disturbances, hypertension, neurocysticercosis or infection, or those with the diagnosis of febrile seizures were excluded from further analysis.

Those children with a diagnosis of epilepsy, as defined by Fisher et al.,^27^ were further assessed for aetiology by reviewing clinical information, laboratory results, brain imaging and EEG studies, if available. In epileptic children diagnosed with HIV encephalopathy with no other clinical, laboratory or imaging findings, the neurotoxic effect of HIV was considered as the cause of epilepsy.

Human immunodeficiency virus encephalopathy is defined as damage or malfunction of the brain because of HIV infection. Human immunodeficiency virus encephalopathy in children must include at least one of the following findings present for at least 2 months in the absence of a concurrent illness other than HIV infection: (1) failure to attain or loss of developmental milestones or loss of intellectual ability; (2) impaired brain growth or acquired microcephaly; or (3) acquired symmetric motor deficit.^28^

The intent of this study was to see how many children in this cohort have epilepsy, even if it was not caused by the HIV infection.

Absolute CD4 count and CD4%, baseline VL at the start of ART, the VL between 6 and 12 months after initiating ART, the VL nearest to the diagnosis of epilepsy (for those diagnosed with epilepsy while on ART and where the value was available within a window of 5 months before or after diagnosis) and VL at last available follow-up assessment were retrieved. Neurological deficits and indicators of developmental delay were also recorded, as well as reports of previous CNS infection. Prescribed antiepileptic drugs were also recorded. Clinical staging of HIV infection as assessed by the attending doctor following the WHO guidelines were documented.

Children attending the clinics were followed up at regular 3-monthly intervals (monthly for children with social problems or adherence issues).

### Ethical consideration

This article followed all ethical standards for a research without direct contact with human or animal subjects.

## Results

Of the 2137 children with confirmed HIV infection enrolled in the two clinics, 53 (2.5%) were diagnosed with epilepsy. Comprehensive medical records were available in 49 (92%) of the 53 patients. Only those children were included in this study. The age of the children ranged from 1 month to 12 years (median 4 years) at the time of diagnosis of epilepsy, and 26 (53%) were boys.

All children and infants with HIV infection and epilepsy were initiated on ART during the study assessment period. Eighty-one per cent of the children were on first-line treatment, which comprised abacavir, lamivudine and lopinavir/ritonavir (*n* = 18) or efavirenz (*n* = 22), depending on the age of the child at initiation and the year of treatment initiation. In 16 of the children, stavudine was replaced by abacavir as part of the change in guidelines for first-line treatment. Four (8%) of the children were placed on lamivudine-holding regimes, mostly for treatment failure because of non-compliance after extensive counselling of the family. The others (11%) were started on second-line treatment following treatment guidelines.

Antiretroviral therapy was started either after (20 patients; 40%), at (15 patients; 31%) or before (14 patients; 29%) diagnosis of epilepsy.

The WHO clinical staging at diagnosis of HIV infection was available in the records for 46 children, with 4 (8%), 5 (10%), 26 (53%) and 11 (23%) children graded at stages 1, 2, 3 and 4, respectively. Staging data were missing in three (6%) children (Table 1).

**TABLE 1:** Clinical staging of HIV infected patients according the World Health Organization guidelines at initiation of treatment.

WHO stage of HIV infection	Number of epileptic children	% of all epileptic patients	Number of children in clinic without epilepsy	% of group
1	4	8	1688	81
2	5	10	84	4
3	26	53	230	11
4	11	23	42	2
Unknown	3	6	42	2

WHO, World Health Organization; HIV, human immunodeficiency virus.

For children who developed epilepsy at the beginning of or after initiating ART (*n* = 29; 59%), the median CD4 count was 591 cells/mm^3^ (range 15 cells/mm^3^ – 1980 cells/mm^3^), and the mean VL was 782 768 copies/mL (range from lower than detectable limit [LDL] to 10 000 000), at the time of diagnosis of epilepsy.

In nine of the 14 children (64%) who were diagnosed with epilepsy while already on ART, the VL was lower than 800 copies/mL or undetectable at the time of epilepsy diagnosis. In four (29%) children, VL was > 800 copies/mL at the time of diagnosis, with one child having no VL data available in the time period. In six (43%) children, the CD4 percentage was < 25%, even though they had been on ART for at least 7 months at the time of diagnosis of epilepsy. Five (36%) children had CD4% > 25%, while CD4% data were not available for three (21%) of the children (Table 2).

**TABLE 2:** Viral loads of patients at diagnosis of epilepsy OR at 6–12 months after initiation of antiretroviral therapy if diagnosis of epilepsy was made before antiretroviral therapy initiation.

VLs	Patients’ VL at time of diagnosis of epilepsy, max 5 months before or after (*N* = 14)		Patients with diagnosis of epilepsy at or before initiation of HIV treatment, VL at 6–12 months after ART initiation (*N* = 35)	
	*n*	%	*n*	%
LDL or < 800 cps/mL	7	50	26	74
> 800 cps/mL	6	43	6	17
Unavailable	1	7	3	9

LDL, lower than detectable levels; HIV, human immunodeficiency virus; VLs, Viral loads.

About 74% (26/35) of the children who were diagnosed with epilepsy before or at ART initiation had a VL that was below the limit of detection or below 800 copies/mL between 6 and 12 months after ART initiation, with only six (17% [6/35]) having VL values > 800 copies/mL. In the group which developed epilepsy at least 7 months after initiation of ART, viral suppression was seen only in 57% (8/14). For three children (9% [3/35]), no data were available.

Nineteen children (39%) were found to have a CNS infection, mainly tuberculosis (*n* = 13), but also other bacterial CNS infections. Three children were diagnosed with neurocysticercosis. Eighteen per cent of the children were diagnosed with HIV encephalopathy by the Centers for Disease Control and Prevention (CDC) criteria,^25^ where neurotoxic effect of HIV was considered as the cause of epilepsy. In one child with spastic quadriplegic cerebral palsy, it was assumed that intra-partum hypoxic ischaemic encephalopathy caused the epilepsy as the child developed seizures right after birth. Two children presenting with cerebrovascular accidents, both caused by persistent severe thrombocytopenia, developed epilepsy thereafter. The average duration between CNS infection and diagnosis of epilepsy was 11 months (range: 1–24 months). In only one case, the diagnosis of epilepsy was made prior to CNS infection. For more than one-third of children (37%), no cause could be found.

Features of neurodevelopmental delay, as assessed by the medical officer in the antiretroviral therapy (ARV) clinic, were present in 17 (34%) of the epileptic children. Several children were referred either for assessment in the neurodevelopmental clinic or to the occupational, physio- or speech therapist. One of the eight children seen in the neurodevelopmental clinic was diagnosed with hemiplegic cerebral palsy post-stroke, one with quadriplegic cerebral palsy because of hypoxic ischemic encephalopathy, two with speech and cognitive impairment of unknown cause and four with HIV encephalopathy.

School failure, school problems, reports from the educational psychologist of intellectual impairment or the notice of attendance of a special school was noted for 27 (55%) of the epileptic children (70% of whom were boys).

Most children were diagnosed with epilepsy before or at the time of diagnosis of HIV infection (35/49). Almost all (48 of 49) children with epilepsy were treated with sodium valproate; 18 received other antiepileptic drugs either before sodium valproate or as a dual- or multi-drug regime, including phenobarbitone (*n* = 16), carbamazepine (*n* = 2), clonazepam (*n* = 2), lamotrigine (*n* = 1) and/or ethosuximide (*n* = 1). The two children treated with carbamazepine were referred from other health facilities on carbamazepine and were changed to sodium valproate in our clinic. Half (24/49) of the children became seizure-free with the use of antiepileptic drugs and eight (16%) had a significant (50% – 75%) reduction in seizure frequency.

## Discussion

In this retrospective survey, we were able to show a 2.5% prevalence of epilepsy in children with HIV infection, which is similar to other areas in South Africa and sub-Saharan Africa, as well as in India.^19,20,21^ This number is about two to three times greater compared to the overall prevalence of epilepsy in Africa and South Africa, which is estimated between 0.73% and 1.2%.^13,28,29^ However, this prevalence might still be underestimated as the data were retrospectively collected from case notes, which is a key limitation of this study.

**FIGURE 1:**
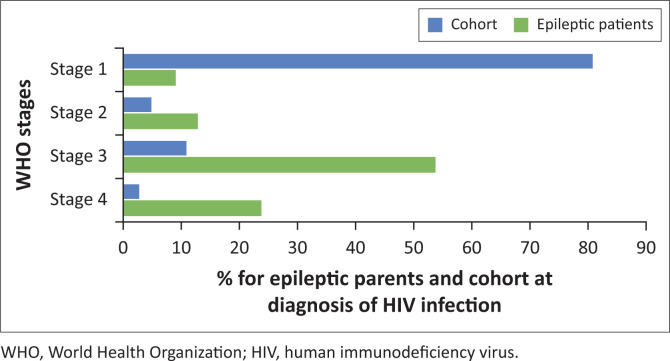
World Health Organization stages in percentage of total number of patients (blue) and in percentage of total number of epileptic patients (green).

Most children (76%) with epilepsy were classified as stage 3 or 4 according to the WHO staging system for HIV/AIDS at diagnosis of HIV. Thus, most of the children with epilepsy were diagnosed at an advanced stage of HIV infection. In contrast, 80% of the children in the group without epilepsy were assessed as stage 1 at diagnosis. (see Table 1 and Figure 1)

In about half of the patients with epilepsy (48%), treatment for HIV infection was initiated at the time of diagnosis. Twenty-eight per cent had treatment initiation months, and sometimes years after HIV diagnosis, probably because of the guidelines for that specific time period. For the rest, the exact date of diagnosis of HIV infection was not available, which is another limitation of this study.

None of the children with epilepsy and stage 1 HIV infection had prior CNS infection, while 81% of the children with epilepsy and stage 4 HIV infection had prior CNS infection. Thirty-seven (75%) of the children were diagnosed with epilepsy before or at initiation of ART. Retrospectively, it was not possible to differentiate in how many children the complaint of seizures was the cause of further investigations, followed by the discovery of HIV infection. These findings might nevertheless indicate not only that many children were only diagnosed as HIV-positive when presenting at the hospital with seizures or CNS infection, but also that some of them did not yet qualify for ART according to the specific HIV treatment guidelines in place at the time. This led to progression of HIV infection in these children and made them vulnerable to opportunistic infections and increased the risk of developing epilepsy. The above findings also support the findings of a prior study,^19^ where early initiation of ART was assumed to be protective for epilepsy and seizures.

The most common specific aetiology for epilepsy was a prior CNS infection, with meningitis caused by Mycobacterium tuberculosis being the most frequent. Again, CNS infection was probably because of late diagnosis of HIV infection, and/or late initiation of ART, making the children vulnerable to opportunistic infections. Human immunodeficiency virus neurotoxicity was the second most common suspected cause. Those data correlate well to those of Bearden et al. in Botswana.^19^ Two children had infection-independent strokes and developed seizures and epilepsy afterwards, and one patient developed seizures as a neonate after suffering from a hypoxic–ischaemic event during birth. As the type of epilepsy was mostly not described in the notes and as most children were unable to receive EEG readings, it is likely that epilepsy syndromes like absence epilepsy or childhood epilepsy with centro-temporal spikes may have been missed. The prevalence of these disorders, however, is low in high resource settings,^30^ but unknown in South Africa. Furthermore, besides CD4 counts and VL, limited laboratory investigations were available for most children, which is a further limitation of this study. In more than one-third of children, the aetiology could not be established, which is slightly higher than that in other studies in children and adults.^19,31,32,33^

In our cohort, almost half of the children with epilepsy became seizure-free and another 16% had a significant reduction (50% – 75%) of seizure frequency. The tight follow-up schedule to assure adherence to ART, and thus to antiepileptic treatment, might have contributed to this outcome.

One big concern is the high percentage of the epileptic children in our cohort with school problems (55%). It is known that many children with perinatal acquired HIV infection show neurodevelopmental delay.^3,15,16,34^ Human immunodeficiency virus-positive preschool and school children had global deficits in all measures of neurodevelopment, except gross motor skills, in a study from Uganda.^35,36,37^ Compared to their non-exposed and non-infected classmates, two to three times more HIV-infected children struggled with language, visual perception and fine motor skills. A study conducted in Congo showed that children infected with HIV had a significantly higher incidence, up to 91%, of neurodevelopmental deficits in all domains compared to uninfected children.^37^ Newer studies could show that early start of treatment with ART in perinatal infected children correlates with higher neuro-cognitive performance in children and adolescents,^38,39,40,41^ supporting the recommendation to preferably start ART in the first 3 months of life.^42,43^

## Conclusion

In this retrospective survey, we were able to show a 2.5% prevalence of epilepsy in infants and children with confirmed HIV infection on ART in a semi-urban–rural part of South Africa. Many of the children who developed epilepsy during the course of their HIV infection had prior CNS infection, or HIV encephalopathy and advanced disease, as demonstrated by the high WHO staging at the start of ART. In addition, more than half (55%) of the children were found to have educational difficulties including referral to a special school.

As shown, HIV-infected infants and children in low-resource settings face an array of additional challenges besides HIV infection, which need to be addressed in a comprehensive manner. It is clear that in a setting with a high prevalence of HIV, early detection of HIV infection and swift initiation of ART in children are crucial, and delays will impact on school performance and thus on future socio-economic status, keeping the spiral of poverty going. The new South African HIV guidelines from 2017, which made it possible to start immediate highly active antiretroviral therapy (HAART) on every person diagnosed with HIV infection, address this issue. Unfortunately, in low-resource areas like the Eastern Cape, early diagnosis in children and infants is still a challenge and this needs to be addressed.
